# Prevalence of early postpartum depression and associated risk factors among selected women in southern Malawi: a nested observational study

**DOI:** 10.1186/s12884-023-05501-z

**Published:** 2023-04-05

**Authors:** E Moya, G Mzembe, M Mwambinga, Z Truwah, R Harding, R Ataide, Leila M Larson, J Fisher, S Braat, SR Pasricha, MN Mwangi, KS Phiri

**Affiliations:** 1 Department of Public Health, School of Global and Public Health, Kamuzu University of Health Sciences, Private Bag 360, Chichiri, BT3, Blantyre, Malawi; 2 Training and Research Unit of Excellence (TRUE), 1 Kufa Road, PO Box 30538, Chichiri, Blantyre BT3 Malawi; 3 Population Health and Immunity Division, The Walter and Eliza Hall Institute of Medical Research, 1G Royal Parade, Parkville, Melbourne, VIC 3052 Australia; 4 The Peter Doherty Institute for Immunity and Infection, Dept of Infectious Diseases, The University of Melbourne, Melbourne, VIC Australia; 5 Department of Health Promotion, Education, and Behavior, Arnold School of Public Health, University of South Carolina, Columbia, SC USA; 6 Global and Women’s Health Unit, School of Public Health and Preventive Medicine, Monash University, Melbourne, VIC Australia; 7 The Health Mothers Healthy Babies Consortium, Micronutrient Forum, 1201 Eye St, NW, 20005-3915 Washington, DC USA

**Keywords:** Anaemia, Delivery, Risk factors, Postpartum depression

## Abstract

**Background:**

The birth of a child should be a time of celebration. However, for many women, childbirth represents a time of great vulnerability to becoming mentally unwell, a neglected maternal morbidity. This study aimed to determine the prevalence of early postpartum depression (PPD) and its associated risk factors among women giving birth at health facilities in southern Malawi. Identifying women vulnerable to PPD will help clinicians provide appropriately targeted interventions before discharge from the maternity ward.

**Method:**

We conducted a nested cross-sectional study. Women were screened for early PPD using a locally validated Edinburgh Postpartum Depression Scale (EPDS) as they were discharged from the maternity ward. The prevalence of moderate or severe (EPDS ≥ 6) and severe (EPDS ≥ 9) PPD was determined, including 95% confidence intervals (CI). Data on maternal age, education and marital status, income source, religion, gravidity, and HIV status, among others, were collected during the second trimester of pregnancy, and obstetric and infant characteristics during childbirth were examined as potential risk factors for early PPD using univariable and multivariable logistic regression analyses.

**Results:**

Data contributed by 636 women were analysed. Of these women, 9.6% (95% CI; 7.4–12.1%) had moderate to severe early PPD using an EPDS cut-off of ≥ 6, and 3.3% (95% CI; 2.1–5.0%) had severe early PPD using an EPDS cut-off of ≥ 9. Multivariable analyses indicated that maternal anaemia at birth (aOR; 2.65, CI; 1.49–4.71, *p*-value; 0.001) was associated with increased risk for moderate and/or severe early PPD, while live birth outcome (aOR; 0.15, 95% CI; 0.04–0.54, *p*-value; 0.004), being single compared to divorced/widowed (aOR; 0.09, 95% CI; 0.02–0.55, *p*-value; 0.009), and lower education level (aOR; 0.36, 95% CI; 0.20–0.65, *p*-value; 0.001) were associated with decreased risk. Being HIV positive (aOR; 2.88, 95% CI; 1.08–7.67, *p*-value; 0.035) was associated with severe PPD only.

**Conclusion:**

The prevalence of early PPD was slightly lower in our selected sample compared to previous reports in Malawi and was associated with maternal anaemia at birth, non-live birth, being divorced/widowed and HIV-positive status. Therefore, health workers should screen for depressive symptoms in women who are at increased risk as they are discharged from the maternity ward for early identification and treatment.

**Supplementary Information:**

The online version contains supplementary material available at 10.1186/s12884-023-05501-z.

## Background

The birth of a baby can be associated with mixed feelings and psychological adjustments. To some women, it is a life-changing moment, one that is bound by love, hope and excitement [1], and represents a period of vulnerability to physical and mental stressors [2]. Globally, the prevalence of postpartum depression (PPD) ranges from 0.5 to 60.8%, and the wide variability in reported PPD prevalence might be due to the use of non-validated screening instruments, lack of consideration of low familiarity with test taking or having low literacy, reporting styles, cross-cultural variables, differences in socio-economic environments (e.g. poverty, levels of social support or its perception, nutrition, stress), differences in perception of mental health and its stigma, and biological vulnerability factors [3]. The prevalence of depression is higher in low and middle-income countries (LMICs) than in high-income countries and it is estimated that depression will be the second-highest cause of disability by 2030 [4]. Depression is described as a psychological state characterised by persistent sadness and a loss of interest in activities that one usually enjoys and is accompanied by an inability to carry out activities of daily living for at least two weeks [1]. Postpartum depression (PPD) is the depression that occurs after childbirth and the current Diagnostic and Statistical Manual for classifying mental disorders (DSM-5) includes peripartum onset specifier [5]. This paper defines depression that occurs within the first week of childbirth as early PPD as previously reported [6]. Of note, some authors have referred to the depression and anxiety symptoms that women experience a few hours after childbirth as maternity blues, a concept characterised by an episodic psychological distress that mimics symptoms of depression and anxiety but is usually self-limiting [7, 8]. However, a recent longitudinal study assessing the transition from maternity blues to full-blown perinatal depression reported that although maternity blues were self-limiting in most of the participants, depressive symptoms arose quite often immediately after childbirth [7].

Untreated PPD can affect the health and survival of both the mother and child. Howard and colleagues described that there is no time in the lifespan that the statement “*there is no health without mental health*” rings truer than in the perinatal period [9]. Additionally, literature has shown that maternal depression in the first weeks after childbirth negatively affects the mother-infant relationship, potentially impacting long-term infant development and health [7, 9]. Additionally, early PPD has been reported as a risk factor for suicidal ideation [11].

The World Health Organization recommends screening for perinatal depression with referral and management services where needed [1]. This recommendation has recently been backed with a global call to action to prioritise perinatal mental health arguing that unless maternal mental health is taken as seriously as their physical wellbeing, maternal morbidity, mortality and the ability of the women to thrive will not be improved [10]. However, it remains unknown whether universal screening for maternal depression after childbirth improves clinical outcomes and whether it is feasible especially in LMICs where human work force is not adequate [6, 12]. In low resource settings, targeted screening is more realistic but it requires the accurate identification of high-risk women. A recent systematic review and meta-analysis of observational studies found a strong association between experiences of violence, unintended pregnancy, caesarian birth, gestation diabetes, and preterm birth with PPD whilst weak association was reported between PPD and postpartum anaemia [14]. However, it is also important to note that the risk factors for PPD vary with women’s age, race, ethnicity and cultural context [15].

Several studies have investigated PPD among women in Malawi. Many of these studies have focused on validating [16, 17] and contextualizing screening tools for PPD and understanding perception of women on perinatal depression [18]. More attention has also been paid to assessing the association between maternal HIV sero-status and postpartum depression than other risk factors [18, 19]. Stewart et al. investigated associated factors for common mental disorder among mothers of infants who were due for measles vaccine, that is at 9 months postpartum or over [21]. However, this study did not collect data on other known risk factors of PPD especially data on events during childbirth and physiological biomarkers for anaemia and iron deficiency. Therefore, our study aimed to determine the prevalence of early postpartum depression and its associated risk factors (socio-demographics, obstetric and clinical factors including maternal anaemia and iron deficiency status at childbirth) among women attending rural and urban health facilities in southern Malawi. Identifying women who are vulnerable to PPD after childbirth will help health practitioners provide appropriate targeted interventions before discharge. We believe that hospitalisation following childbirth provides opportunity for screening and treatment of early PPD as it is a time when women are in touch with health services.

## Methods

### Study design and sample size

We conducted a cross-sectional study nested in two ongoing studies (1) the Randomised controlled trial of Effect of intraVenous iron on Anaemia in Malawian Pregnant women (REVAMP study) [22], and (2) an observational cohort (REVAMP Observe). Based on previous estimates of the prevalence of depression (13.9% and 11.0%) among postpartum women attending child health services at health facilities in southern Malawi [18, 20], we assumed the prevalence of PPD to be 13%. A minimum sample size of 179 was required to obtain a two-sided 95% confidence interval of +/- 5% (i.e. 95% CI; 8–18%) using a Wald confidence interval.

### Participants and study sites

**Fig. 1:**
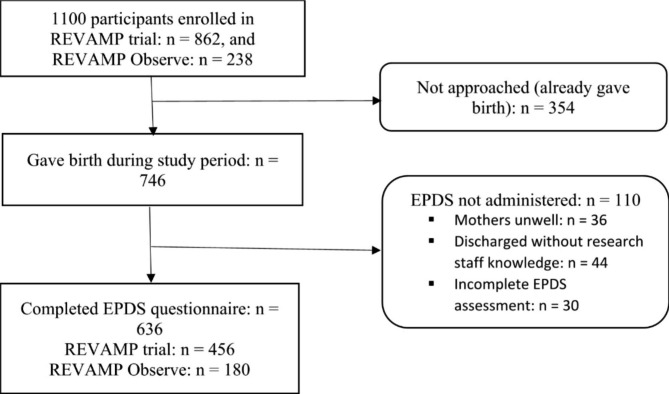
Flow chart showing enrollment of participants from REVAMP trial and REVAMP Observe. (EPDS: Edinburg Postpartum Depression Scale, REVAMP: Randomised control trial of Effect of intraVenous iron on anaemia in Malawian Pregnant women. The participants were recruited in their second trimester (gestation age between 13 and 26 weeks)

The participants were a cohort of women who gave birth at urban and rural health centres within Zomba and Blantyre districts, southern Malawi and were part of the REVAMP and REVAMP Observe studies. Details of the inclusion and exclusion criteria for the REVAMP trial have been published elsewhere [22]. Briefly, the REVAMP trial recruited 862 anaemic (haemoglobin level ≥ 5 but < 10 g/dl) pregnant women in their second trimester and randomised them to either standard of care treatment of oral ferrous sulphate (200 mg twice a day for 90 days or until birth whichever comes first) for the remainder of pregnancy or intravenous ferric carboxymaltose (20mgs/kg or maximum of 1000 mg for ≥ 50 kg) once. The REVAMP observe study had similar inclusion and exclusion criteria to the REVAMP trial except that their baseline haemoglobin level was ≥ 10 g/dl (half between 10 and 11 g/dl and the other half > 11 g/dl) and women were not given any study intervention. However, participants from REVAMP Observe might have received oral ferrous sulphate with folic acid from government health facilities during their antenatal visits as this is part of routine care. As part of our nested study, women who gave birth between December 2019 and August 2021 were approached for postpartum depression assessment (Fig. 1) as they were discharged from the maternity ward. By that time, the women who had consented to be followed-up in the two parent studies were expected to still come for their subsequent follow-up visits. The potential impact of addressing anaemia on depression was not discussed with women prior to enrolment.

### Study measurements and procedures

#### Socio-demographic and obstetric variables

Data for maternal socio-demographic variables and obstetric history such as age, education status, marital status, religion, source of income, maternal HIV status, history of miscarriage, gravidity and smoking history were derived from the REVAMP and REVAMP Observe databases. The data were collected when these women were enrolled into the REVAMP and REVAMP Observe studies during the second trimester of their pregnancy.

#### Pregnancy and neonatal outcome data

Trained research nurses collected data on pregnancy outcomes which included data on whether a woman gave birth spontaneously, assisted or by cesarean, place of childbirth, estimated blood loss with postpartum hemorrhage defined as blood loss of ≥ 500mls estimated visually by midwives, childbirth complications (i.e. cord prolapse, perineal tear, abnormal fetal heart rate, obstructed labour, premature rupture of membrane), whether the woman received blood transfusion after childbirth and pregnancy outcome categorised as full term (≥ 37 weeks’ gestation age), preterm (< 37 weeks’ gestation age), still birth (baby born dead after 28 completed weeks of pregnancy) and spontaneous and induced abortion (baby born dead before 28 completed weeks of pregnancy). For the newborn, a trained research midwife collected data through a structured questionnaire on whether the baby suckled immediately after birth, birth weight, sex of the baby and whether the baby was admitted immediately after birth.

#### Postpartum depression

Maternal depression status was assessed using a Chichewa translated and validated Edinburgh Postpartum Depression Scale (EPDS) on discharge from the maternity ward, between 24 and 72 h after childbirth. The EPDS is a 10-item self-report scale and each question is rated on a scale of 0 to 3. Total scores can range from 0 to 30, with a higher score representing more depressive symptoms. The Chichewa EPDS version has shown to be a valid tool for screening and detecting episodes of both major and minor depression among women who are pregnant (AUC = 0.77, 95% CI; 0.69–0.84) in Malawi. When compared with the gold standard Structured Clinical Interview for DSM-IV (SCID) for major depression, the Chichewa EPDS version demonstrated a specificity of 74.1% and sensitivity of 76.3% at a cut-off 5/6 [23]. However, a previous multi-site study in similar settings used a higher EPDS score cut-off of ≥ 9 [24]. Therefore, we chose to use both cut-off points to make our findings comparable with findings from both local and international studies. Trained research nurses and midwives administered the EPDS questionnaire to all study participants on discharge from the maternity ward. The interviews were conducted in a closed room at our research site within the health facilities’ premises.

#### Blood sampling

Trained research nurses collected finger prick capillary blood and venous blood from the mothers at childbirth. After wiping off the first drop, the second drop of finger prick blood was immediately used for checking haemoglobin concentrations and malaria falciparum rapid test. We used the Hemocue 301+ (Angelholm, Sweden) which has an inbuilt system for internal quality control to measure haemoglobin concentrations and CareStart™ Malaria HRP2/Pldh, Pf/Pan (Access Bio, Somerset, NJ 08873, USA) for rapid diagnoses of malaria (mRDT). Maternal anaemia at childbirth was defined as a haemoglobin (Hb) concentration of < 11 g/dl [25]. We used the Hemocue 301 + Hb and mRDT results for anaemia and malaria classification respectively as it is the current practice in most of the health facilities in Malawi.

Trained laboratory technologists separated serum from whole blood and stored samples in a -80^o^C freezer before shipping them to the Netherlands for analysis of serum ferritin levels and the inflammatory marker, C-reactive protein (CRP). The analysis was done at Abbott Architect IA analyser at Meander Medical Centre, Amersfoort, The Netherlands, with reagents from and as per instructions of the manufacturer (Architect IA Ferritin Reagent). Iron deficiency was defined as ferritin < 15 µg/l or ferritin < 30 µg/l if CRP > 5 mg/l and iron deficiency anaemia referred to Hb levels < 11 g/dl (Sysmex haematology analyser) and iron deficiency as above. Presence of inflammation was defined as CRP values > 5 mg/L [25].

### Statistical analysis

Maternal socio-demographic variables (age, education level, marital status, religion and source of income), obstetric characteristics at birth (gravidity, miscarriage history, pregnancy outcome, mode of childbirth, birth complication, anaemia, malaria status, HIV status, iron deficiency, iron deficiency anaemia, inflammation), newborn characteristics (sex, birth weight and whether the newborn was admitted at nursery) were summarised and presented as counts and percentages. The prevalence of moderate or severe (EPDS ≥ 6) and severe (EPDS ≥ 9) PPD, based on maternal depression status at discharge from maternity ward (between 24 and 72 h after childbirth depending on maternal condition), and corresponding Clopper-Pearson two-sided 95% confidence intervals (CI) were obtained.

We used univariable logistic regression to assess the association between early PPD, defined as an EPDS score ≥ 6 (moderate or severe) and ≥ 9 (severe), and all the study variables. We ran a multivariable analysis to examine risk factors of moderate or severe depression including only variables with a *p*-value ≤ 0.20 in the univariate models. Due to the small number of women with severe depression (EPDS ≥ 9, *n* = 21), we only included variables with a *p*-values < 0.05 (univariable analyses) in the multivariable logistic regression model to examine risk factors of severe depression. Not all measurements were complete for some demographic and clinical data and in such a case statistical analyses were done on the actual available data. We chose the binary version of the variables such as hemoglobin (anaemic and non-anaemic) and not the continuous version (haemoglobin levels) for fitting in the model as it has an easier clinical interpretation. In order to test for multicollinearity, we checked whether the variables of the multivariable model (moderate or severe depression) were strongly correlated with each other (e.g., “pregnancy outcome” and “birth weight”). For severe depression model, the check was performed for variables “anaemia” and “HIV status”. Data analyses were performed using Stata SE 15.1 (STATA Corp, College Station, TX, USA).

### Ethical consideration

The present study was a sub-study embedded in two studies. The REVAMP trial was approved by the College of Medicine Research and Ethics Committee (COMREC), Malawi (P.02/18/2357) and the Walter and Eliza Hall Institute of Medical Research Ethics Committee, Australia (WEHI REC 18/02). The REVAMP Observe study was approved by COMREC (P.02/20/2951). Information about the substudy was given to all study participants, as they were enrolled in the main studies, and all participants either signed a consent form or printed a thumb print (observed by an impartial witness) for those who could not write. All participants were also informed about voluntary participation, data confidentiality and their right to withdraw from participating at any study point. Participants who were identified as severely depressed were referred to the local Ministry of Health psychiatric services for further assessment and treatment.

## Results

### Characteristics of the study participants

Of the 746 women who gave birth during the study period, a total of 636 (85.3%) participants completed EPDS assessments for this substudy (Fig. 1). The demographic variables between those who completed the EPDs questionnaire did not differ statistically from those who did not (data not shown). A total of 456 (71.8%) and 180 (28.2%) were enrolled from REVAMP trial and REVAMP observe study respectively. The respondents’ age ranged between 15 and 41 years with a mean (± SD) age of 22.8 ± 6.4 years. Detailed socio-demographic characteristics of the study participants during pregnancy has been presented in Table 1.

**Table 1:** Characteristics of the study participants during second trimester (gestation age by ultrasound 13-26 weeks)

		Maternal early PPD status			
		EPDS ≥6		EPDS≥9	
	Total	Yes (*n*=61)	No (*n* =575)	Yes (*n* =21)	No (*n* =615)
Maternal age (*n* =634)					
≥20 years old <20 years old	366 (57.7%) 268 (42.3%)	41 (11.2%) 20 (7.5%)	325 (88.8%) 248 (92.5%)	14 (3.8%) 7 (2.6%)	352 (96.2%) 261 (97.4%)
Education status (*n* =629)					
Secondary school or more Primary school or less	209 (33.2%) 420 (66.8%)	30 (14.3%) 30 (7.1%)	179 (85.7%) 390 (92.9%)	8 (3.8%) 13 (3.1%)	201 (96.2%) 407 (96.9%)
Marital status (*n* =636)					
Divorced/widow Never married/single Married/cohabiting	12 (1.9%) 98 (15.4%) 526 (82.7%)	3 (25.0%) 4 (4.1%) 54 (10.3%)	9 (75.0%) 94 (95.9%) 472 (89.7%)	2 (16.7%) 2 (2.0%) 17 (3.2%)	10 (83.3%) 96 (98.0%) 509 (96.8%)
Source of income (*n* =636)					
Casual work Farming Permanent work Business	227 (35.6%) 155 (24.4%) 85 (13.4%) 169 (26.6%)	26 (11.5%) 13 (8.4%) 6 (7.1%) 16 (9.5%)	201 (88.5%) 142 (91.6%) 79 (92.9%) 153 (90.5%)	8 (3.5%) 5 (3.2%) 2 (2.4%) 6 (3.6%)	219 (96.5%) 150 (96.8%) 83 (97.6%) 163 (96.4%)
Religion (*n* =636)					
Non-Christian Christian	153 (23.9%) 483 (76.1%)	14 (9.2%) 47 (9.7%)	139 (90.8%) 436 (90.3%)	5 (3.3%) 16 (3.3%)	148 (96.7%) 467 (96.7%)
Gravidity (*n* =635)					
Multi-gravida Primi-gravida	324 (51.0%) 311 (49.0%)	35 (10.8%) 26 (8.4%)	289 (89.2%) 285 (91.6%)	12 (3.7%) 9 (2.9%)	312 (96.3%) 302 (97.1%)
History of miscarriage (*n* =635)					
No Yes	591 (93.1%) 44 (6.9%)	58 (9.8%) 3 (6.8%)	533 (90.2%) 41 (93.2%)	19 (3.2%) 2 (4.6%)	572 (96.8%) 42 (95.5%)
Maternal HIV status (*n* =636)					
Sero-positive Sero-negative	91 (14.3%) 545 (85.7%)	10 (11.0%) 51 (9.4%)	81 (89.0%) 494 (90.6%)	7 (7.7%) 14 (2.6%)	84 (92.3%) 531 (97.4%)

EPDS: *Edinburgh Postpartum Depression Scale; PPD = Postpartum depression*

Gravidity: *sum of all pregnancies, including all live births and pregnancies terminated or did not result in a live birth (multi-gravida; ever had more than one pregnancies, primi-gravida; first pregnancy)*

Early PPD: *postpartum depression measured between 24 to 72 h after child birth*

Table 2 presents maternal and infant characteristics at birth. A total of 611 (96.7%) women had a live birth and 21 (3.3%) women had either a stillbirth or miscarriage. Four women had their record on pregnancy outcome missing. A total of 323 (50.9%) newborns were male and 89 (14.0%) had low birth weight (< 2500 g). Only 28 (4.4%) women had malaria by rapid test and 161 (25.3%) were anaemic at delivery. Assessments for iron deficiency, iron deficiency anaemia and inflammation were performed on the REVAMP trial participants only and results for 440 (69.2%) women were available for analysis. Iron deficiency adjusted for inflammation was found in 124 (28.2%) women whilst iron deficiency anaemia in 54 (12.3%) women. Elevated CRP (a sign of acute inflammation) was noted in 319 (72.5%) women and anaemia and inflammation in 93 (21.2%).

**Table 2:** Maternal and infant characteristics during child birth

		Maternal early PPD status			
		EPDS ≥6		EPDS≥9	
	Total	Yes (*n* =61)	No (*n* =575)	Yes (*n* =21)	No (*n* =615)
Pregnancy outcome (*n* =632)					
Poor pregnancy outcome Good pregnancy outcome	21 (3.3%) 611 (96.7%)	6 (28.6%) 55 (9.0%)	15 (71.4%) 556 (91.0%)	5 (23.8%) 16 (2.6%)	16 (76.2%) 595 (97.4%)
Mode of birth (*n* =632)					
Vaginal birth Caesarian section	559 (88.4%) 73 (11.6%)	54 (9.7%) 7 (9.6%)	505 (90.3%) 66 (90.4%)	20 (3.6%) 1 (1.4%)	539 (96.4%) 72 ((98.6%)
Complication at childbirth (*n* =632)					
No Yes	495 (78.3%) 137 (21.7%)	51 (10.3%) 10 (7.3%)	444 (89.7%) 127 (92.7%)	20 (4.0%) 1 (0.7%)	475 (96.0%) 136 (99.3%)
Postpartum hemorrhage (*n* =612)					
No Yes	598 (97.7%) 14 (2.3%)	57 (9.5%) 2 (14.3%)	541 (90.5%) 12 (85.7%)	21 (3.5%) 0 (0.0%)	577 (96.5%) 14 (100%)
Sex of newborn (*n* =634)					
Female Male	311 (49.1%) 323 (50.9%)	28 (9.0%) 33 (10.2%)	283 (91.0%) 290 (89.8%)	7 (2.3%) 14 (4.3%)	304 (97.8%) 309 (95.7%)
Birth weight (*n* =634)					
≥2500 g (normal birth weight) <2500 g (low birth weight)	545 (86.0%) 89 (14.0%)	49 (9.0%) 12 (13.5%)	496 (91.0%) 77 (86.5%)	16 (2.9%) 5 (5.6%)	529 (97.1%) 84 (94.4%)
Newborn admitted (*n* =635)					
No Yes	558 (87.9%) 77 (12.1%)	55 (9.6%) 6 (10.0%)	520 (90.4%) 54 (90.0%)	19 (3.3%) 2 (3.3%)	556 (96.7%) 58 (96.7%)
Maternal malaria status (*n* =633)					
Malaria negative Malaria positive	605 (95.6%) 28 (4.4%)	59 (9.9%) 2 (7.1%)	545 (90.1%) 26 (92.9%)	21 (3.5%) 0 (0%)	584 (96.5%) 28 (100%)
Maternal anaemia status (*n* =636)					
Non- anaemic (Hb≥11 g/dl) Anaemic (Hb <11 g/dl)	475 (74.7%) 161 (25.3%)	36 (7.6%) 25 (15.5%)	439 (92.4%) 136 (84.5%)	11 (2.3%) 10 (6.2%)	464 (97.7%) 151 (93.8%)
Maternal ID status (*n* =440)					
Non ID ID	316 (71.8%) 124 (28.2%)	20 (6.3%) 12 (9.7%)	296 (93.7%) 112 (90.3%)	9 (2.9%) 3 (2.4%)	307 (97.1%) 121 (97.6%)
Maternal IDA status (*n* =439)					
Non IDA IDA	385 (87.7%) 54 (12.3%)	6 (11.1%) 26 (6.8%)	48 (88.9%) 359 (93.2%)	2 (3.7%) 10 (2.6%)	52 (96.3%) 375 (97.4%)
Maternal inflammation (*n* =440)					
No Inflammation Inflammation	121 (27.5%) 319 (72.5%)	5 (4.1%) 27 (8.5%)	116 (95.9%) 292 (91.5%)	1 (0.8%) 11 (4.5%)	120 (99.2%) 308 (96.5%)

Early PPD: *postpartum depression measured between 24 to 72 h after child birth*

EPDS: *Edinburgh Postpartum Depression Scale; PPD = Postpartum depression*

ID: *iron deficiency indicated as serum ferritin<15 ug/L or ferritin <30 ug/L if C-reactive protein >5 μg/L*

IDA: *iron deficiency anaemia indicated as Hb<11 g/dL and serum ferritin<15 ug/L or ferritin <30 ug/L if C-reactive protein >5 μg/L*

Inflammation: *indicates C-reactive protein >5μg/L*

Good pregnancy outcome: *live birth either full term or preterm birth*

Poor pregnancy outcome: *stillbirth, spontaneous abortion, elective or induced abortion*

Approximately 9.6% (95% CI; 7.4–12.1%) and 3.3% (95% CI; 2.1–5.0%) had early PPD at EPDS cut-off ≥ 6 and ≥ 9 respectively at discharge from the maternity ward (between 24 and 72 h after childbirth). The total individual EPDS score ranged from 0 to 14 and the median (interquartile range) was 1 (0–3). A total of 227 (36.1%) women had a zero EPDS score of which 159/227 (70.0%) had primary school or less education.

### Risk factors for early PPD

In univariable analyses, maternal anaemia at birth (OR; 2.24, 95% CI; 1.30–3.87, p value = 0.004) increased the odds of early PPD whilst live birth (OR; 0.25, 95% CI; 0.09–0.66, p value = 0.006), being single/never married compared to being divorced/widowed (OR; 0.13, 95% CI; 0.02–0.66, *p*-value = 0.014) and lower education status (OR; 0.46, 95% CI; 0.27–0.78, *p*-value = 0.004) decreased the odds of early PPD using an EPDS cut off of ≥ 6 (Table 3). No evidence of an association was found for iron deficiency (OR; 0.85, 95% CI; 0.23–3.18, *p*-value = 0.80), iron deficiency anaemia (OR; 1.44, 95% CI; 0.31–6.77, *p*-value = 0.64), and other variables as potential risk factors (Supplementary Table 1). In multivariate analysis, the observed association remained significant for anaemia (adjusted OR; 2.65, 95% CI; 1.49–4.71, *p*-value = 0.001), being single/never married compared to being divorced/widowed (adjusted OR; 0.09, 95% CI; 0.02–0.55, *p*-value = 0.009), live birth (adjusted OR; 0.15, 95% CI; 0.04–0.54, *p*-value = 0.004) and lower education status (adjusted OR; 0.36, 95% CI; 0.20–0.65, *p*-value = 0.001) (Table 3). Pregnancy outcome was not strongly correlated with low birth weight in our sample, therefore both were maintained in the model.

**Table 3:** Univariable and multivariable odds ratios of potential risk factors for early postpartum depression (EPDS ≥6) at rural and urban health facilities in Zomba and Blantyre, Malawi (*n* = 622)

Characteristics	Crude odds ratio (95% CI)	*p*-value	Adjusted odds ratio (95% CI)	*p*-value
Maternal age				
≥ 20 years old < 20 years old	*ref* 0.64 (0.37 - 1.12)	0.12	*ref* 0.93 (0.50 -1.70)	0.81
Education level				
Secondary school or more Primary school or less	*ref* 0.46 (0.27 - 0.78)	0.004	*ref* 0.36 (0.20 - 0.65)	0.001
Marital status		0.01		0.19
Divorced/widow Never married/single Married/cohabiting	*ref* 0.13 (0.02 - 0.66) 0.34 (0.09 - 1.31)	0.014 0.12	*ref* 0.09 (0.02 - 0.55) 0.26 (0.06 - 1.05)	0.009 0.059
Pregnancy outcome				
Poor outcome Good outcome	*ref* 0.25 (0.09 - 0.66)	0.006	*ref* 0.15 (0.04 - 0.54)	0.004
Maternal anaemia status				
Non-anaemic (Hb≥11 g/dl) Anaemic (Hb <11 g/dl)	*ref* 2.24 (1.30 - 3.87)	0.004	*ref* 2.65 (1.49 - 4.71)	0.001
Birth weight				
≥2500 g (normal birth weight) <2500 g (low birth weight)	*ref* 1.58 (0.80 - 3.10)	0.19	*ref* 0.97 (0.39 - 2.44)	0.95

CI: *confidence interval*

Good outcome: *live birth either full term or preterm birth*

Poor outcome: *stillbirth, spontaneous abortion, elective or induced abortion*

ref: *reference group*

*n* =622: *complete case analysis of the multivariable model*

Using an EPDS cut-off of ≥ 9, univariable analyses showed variables maternal HIV positive status (OR; 3.16, 95% CI; 1.24–8.06, *p*-value = 0.016), and maternal anaemia at delivery (OR; 2.79, CI; 1.16–6.71, *p*-value = 0.022) were significant risk factors whilst live birth (OR; 0.09, 95% CI; 0.03–0.26, *p*-value; <0.001) conferred protection for early PPD. All these variables remained significantly associated with early PPD in a multivariable analysis (Table 4). Anaemia was not strongly correlated with HIV sero-positivity of the mother in our sample, therefore both were maintained in the model.

**Table 4:** Univariable and multivariable odds ratios of potential risk factors for early postpartum depression (EPDS ≥9) at rural and urban health facilities in Zomba and Blantyre, Malawi (*n* = 622)

Characteristics	Crude odds ratio (95% CI)	*p*-value	Adjusted odds ratio (95% CI)	*p*-value
Maternal HIV status				
Sero-negative Sero-positive	*ref* 3.16 (1.24 - 8.06)	0.016	*ref* 2.88 (1.08 - 7.67)	0.035
Pregnancy outcome				
Poor outcome Good outcome	*ref* 0.09 (0.03 - 0.26)	<0.001	*ref* 0.07 (0.02 - 0.24)	<0.001
Maternal anaemia status				
Non-anaemic (Hb≥11 g/dl) Anaemic (Hb <11 g/dl)	*ref* 2.79 (1.16 - 6.71)	0.022	*ref* 3.03 (1.20 - 7.65)	0.019

CI: *confidence interval*

Good outcome: *live birth either full term or preterm birth*

Poor outcome: *stillbirth, spontaneous abortion, elective or induced abortion*

Ref: *reference group*

*n* =622: *complete case analysis on multivariable model*

## Discussion

The present study examined the prevalence and associated risk factors for early PPD among women who were enrolled in REVAMP and REVAMP Observe studies and gave birth at rural and urban health facilities in southern Malawi. We find that anaemia at delivery increased the risk for moderate and/or severe depression whilst HIV positive status increased the risk for severe depression. Other factors, such as lower education status (primary school or less education) and giving birth to live infants, reduced the risk for early PPD among the study participants. We found the prevalence of early PPD to be slightly lower than what has been previously reported in our setting.

### The prevalence of early PPD

The prevalence estimates of early PPD in this study, which likely included maternity blues and depression persisting from pregnancy, was 9.6% (95% CI; 7.4–12.1%) and 3.3% (95% CI; 2.1–5.0%) using an EPDS cut-off score of ≥ 6 and ≥ 9 respectively. Variations in study design, population studied (enrolling only HIV-positive women), different study points at which PPD was measured and studies using different screening tools and cut-off scores for classifying postpartum depression make direct comparison of the reported prevalence among studies almost impossible. However, a recent multi-country cross-sectional study determining physical and psychological comorbidities in Malawi, India, Pakistan and Kenya reported higher early PPD prevalence (defined using an EPDS cut-off of ≥ 10 within 1–7 days after childbirth) in Malawi than what we found. Authors in this study reported the prevalence of early PPD as 15.0% [5]. The most likely reason for the observed difference could be the strict inclusion and exclusion criteria of the two studies from where our study participants were drawn. It is also important to note that, historically malaria has been linked to mood disorders [26], and few women in this study had peripheral P. falciparum parasitaemia detected by rapid diagnostic test (Supplementary Table 1) at childbirth. The low malaria prevalence might be a result of a direct observed Intermittent Preventive Treatment in pregnancy (IPTp) with SP, 1500 mg sulfadoxine and 75 mg pyrimethamine that women received and the insecticide treated bed nets that were given as part of the two parent studies.

### Risk factors for early PPD

Postpartum depression is a complex phenomenon that includes interaction between biochemical, genetic, economic, psychosocial and situational life stress factors. The risk factors for postpartum depression have predominantly included psychosocial factors with weak evidence for other physiological factors such as anaemia and mode of birth (cesarean section) [14]. Our study assessed associated risk factors (i.e. maternal socio-demographic and obstetric characteristics during second trimester, obstetric, maternal and infant characteristics) for early PPD.

Regarding maternal socio-demographic variables, our study findings indicated a significant association between education level, marital status and early PPD. Women who only attended primary school or less were less likely to experience symptoms of early PPD than those who went beyond primary education. Our findings are in conflict with several studies that have reported maternal lower education status as a risk factor for PPD [27, 29, 30]. It is important to note that 227 (36.1%) participants in our study had a total individual EPDS score of zero, of which 159/227 (70.0%) were women with lower education. The findings suggested that women with lower education in our study had a tendency of rating items on the scale as zero possibly because they did not understand or were not familiar with the rating scale. This was despite that trained nurses administered the EPDS questionnaire. Additionally, it has been speculated that women with lower education status and mostly poor do not regard psychological problems as health problems and mostly because of fear of being stigmatized they do not disclose their mental illness to others [11]. However, we do not think that our study participants had these fears as the interviews were done in private rooms and the interviewers had already met and interacted with the participants at several study points as part of the main studies.

Our findings on marital status are consistent with several studies that have reported being divorced/widowed as a risk factor maternal PPD [28, 30]. However, it is important to note that some factors that are connected to marital status and known risk factors for PPD such as family violence, poverty or wealth, dowry or bride price were not assessed in our study thereby limiting comparison with previous research. Our study did not find significant associations between other socio-demographic factors such as age, source of income and religious beliefs.

Our study found HIV-positive status as a significant risk factor for the severe form of early PPD. This finding was expected considering that women living with HIV are reported to have high prevalence of physical and sexual abuse, caregiving stress and elevated internalized stigma [31]. Previous studies in Malawi have reported mixed findings on whether maternal HIV-positive status is a risk factor for PPD. For example, Stewart et al. [21], Zafar et al. [24], and Harrington et al. [20] reported a significant association between maternal HIV-positive status and PPD while Dow et al. [19] reported no association between maternal HIV status and PPD. Possible explanations for these mixed findings may be the use of different screening tools and, although Zafar et al. and Dow et al. used EPDS, they used different cut-off points for depression classification. However, future research should further explore on the reasons for these inconsistent findings, as our study also found no association with PPD using an EPDS score cut-off of ≥ 6.

Inconsistent findings have been reported on some obstetric variables such as gravidity/parity, history of miscarriage, pregnancy outcome, mode of childbirth, birth complications and postpartum haemorrhage (PPH) as risk factors for PPD [32]. Several studies have reported that primiparous women are at increased risk for PPD [35–36] whilst other authors have reported no significant association between being primigravid and the increased risk for PPD [35]. Our study finding is consistent with those studies that reported primigravid as a non-significant risk factor for PPD. Although authors who found a significant association between the two argued that first time mothers have increased fear during pregnancy and at childbirth predisposing them to the development of depressive symptoms [34], we believe that with adequate support from family members or others [29], these fears might be relieved. This study did not find history of miscarriage, mode of birth, childbirth complication, and PPH as significant risk factors for early PPD as reported in previous studies. Poor pregnancy outcome (still birth and experiencing miscarriage in the current pregnancy) was a risk factor for both moderate and severe PPD in our study. This finding is consistent with several other studies [30, 37, 38, 39]. However, considering the time at which EPDS was administered, it could be argued that the symptoms observed were a result of a normal grieving process and not necessarily depressive symptoms.

None of the newborn characteristics that were measured in this study were found to be significant risk factors for early PPD. We found no evidence of associations between sex of the newborn, low birth weight, immediate admission of the newborn and early PPD. In some cultures where sex of the newborn is a preference, female sex has been reported as a risk factor for early PPD [39]. Some authors have also reported very low birthweight or preterm birth as risk factor for PPD [39]. Our study did not further classify the low birthweight which might obscure the possible association. However, it is important to note that previous studies have also reported no association between newborn sex [40], birth weight [29] and PPD, which is consistent with our findings.

Maternal anaemia at childbirth increased the risk for both moderate and severe early PPD. Although our findings are consistent with several individual studies and results from meta-analysis and systematic reviews [42, 43], several observation studies found no association between the two. For example, Armony-Sivan et al. found no relationship between either maternal Hb levels or iron status and PPD [43]. The authors in this study reported no significant difference in EPDS scores between anaemic and non-anaemic mothers regardless of timing of maternal Hb and iron assessments. In addition, the authors reported that women with or without PPD had similar iron status [43]. Another study also reported no significant difference in hemoglobin or iron status in women who had PPD compared to those without (OR: 0.69, 95% CI: 0.15–2.49) [44]. However, this study had high loss to follow-up as only 103/248 were assessed at 6 weeks and none of the women were anaemic at the time of EPDS assessment thereby limiting the variability in the exposure. A recent umbrella review of the current evidence from systematic reviews and meta-analyses of observational studies on risk factors of postpartum depression has reported a weak association between maternal anaemia and postpartum depression [14].

Neither iron deficiency nor iron deficiency anaemia were found to be associated with early PPD in this study. Our findings are consistent with a previous longitudinal study conducted in China [43], but in conflict with several other studies [45]. Iron is a crucial cofactor in the synthesis of serotonin, dopamine and norepinephrine in the brain, neurotransmitters implicated in clinical depression [46]. Furthermore, iron is an essential element in the production of hemoglobin, and iron supplementation increases haemoglobin [47]. Low haemoglobin levels in the body cause fatigue that might lead to depression [48]. In this study, we classified iron deficiency based on serum ferritin levels adjusting for inflammatory markers (CRP). Elevated CRPs is an indication of acute inflammatory process and not a chronic inflammatory process which might be associated with pregnancy [23], limiting its accuracy during this period. Further studies should determine possible association between these two conditions at childbirth using iron markers that are not affected by inflammation such as hepcidin or soluble transferrin receptors.

### Study limitations

Our study has several limitations. First, maternal depression status was not measured using a gold standard diagnostic approach and the measurement time may have led to maternity blues being misinterpreted as PPD. We also acknowledge the emotional disruption (focus shift to wellbeing of the baby and not on women themselves) that occurs in the early postpartum days. However, we used a locally validated EPDS tool that demonstrated good internal and construct validity against a structured clinical interview for DSM-IV [23] and the recent DSM-V includes peripartum onset as a qualifier for postpartum depression [4], which is within our assessment time. Second, our participants are not a true representation of the population of pregnant women in Malawi as they were drawn from other studies that had strict inclusion and exclusion criteria. Third, we did not collect data on other important risk factors for PPD such as hormonal levels, social support, household wealth and gender-based violence [48] thereby limiting generalisability of the study findings. Nevertheless, this is the first study to our knowledge in Malawi that has examined anaemia, iron deficiency, iron deficiency anaemia and inflammation at childbirth as potential risk factors for early PPD.

### Clinical implications

Our findings have several clinical implications. First, depressive symptoms (moderate or severe PPD) is prevalent in women immediately after childbirth and identifying women with depression at an earlier stage might reduce the severity and chronicity of the condition. This is important considering that early maternal PPD has been associated with increased risk for maternal suicidal ideation [9], and poor child growth and development. Second, our study has identified potential risk factors for early PPD which clinicians can pay more attention to if routine screening for PPD is not feasible, a common case in LMICs. Previous studies have shown that routine screening for PPD is not feasible in Malawi due to human resource constraints. Therefore, our study findings can help health practitioners to target at-risk populations and provide appropriate therapy or referral. These at-risk populations include; HIV sero-positive women, women who gave birth to non-live infants, women with unstable marital status, and those with anaemia during childbirth.

## Conclusion

Our findings indicate a slightly lower prevalence of early PPD among women who gave birth at health facilities in southern Malawi than previously reported and was associated with anaemia at childbirth, non-live birth, being divorced/widowed and HIV positive status. Given that most of the women in Malawi give birth at health facilities, health practitioners should screen for depressive symptoms in women who are at increased risk (if routine screening is not feasible) as they are discharged from the maternity ward for early identification and treatment.

## Electronic supplementary material

Below is the link to the electronic supplementary material.

**Additional file 1. Supplementary Table 1.** Univariable odds ratios of potential risk factors for early postpartum depression at rural and urban health facilities in Zomba and Blantyre, Malawi

## Data Availability

The datasets generated and/or analysed during the current study are not publicly available. However, it can be accessible from the corresponding author upon request.

## Abbreviations

aOR: adjusted Odds Ratio
CI: Confidence Interval
CRP: c-reactive protein, an inflammatory marker
DSM: Diagnostic and Statistical Manual for diagnosing mental disorders
EPDS: Edinburgh Postpartum Depression Scale
Hb: Haemoglobin
HIV: Human immunodeficiency virus
LMICs: Low and Middle Income Countries
PPD: Postpartum Depression
REVAMP: Randomised control trial on Effect of intraVenous iron on Anaemia in Malawian Pregnant women
SCID: Structured Clinical Interview for DSM-V
SD: Standard Deviation
