# Supplementary material for: Prevalence of early postpartum depression and associated risk factors among selected women in southern Malawi: a nested observational study
Source: BMC Pregnancy Childbirth. 2023 Apr 5;23:229. doi: 10.1186/s12884-023-05501-z (PMC10074867; doi:10.1186/s12884-023-05501-z)
Supplement: Supplementary file 1 — Additional file 1. Supplementary Table 1. Univariable odds ratios of potential risk factors for early postpartum depression at rural and urban health facilities in Zomba and Blantyre, Malawi [file 12884_2023_5501_MOESM1_ESM.docx]

| Supplementary Table 1: Univariable odds ratios of potential risk factors for early postpartum depression at rural and urban health facilities in Zomba and Blantyre, Malawi | | | | |
| --- | --- | --- | --- | --- |
| Characteristics | Crude odds ratio (95% CI) | P-value | Crude odds ratio (95% CI) | P-value |
|  | EPDS ≥6 (n=61) | | EPDS ≥9 (n=21) | |
| During second trimester (gestation age by ultrasound 13-26 weeks) | | | | |
| Maternal age (n=634)  ≥ 20 years old  < 20 years old | *ref*  0.64 (0.37 - 1.12) | 0.12 | *ref*  0.67 (0.27 - 1.69) | 0.40 |
| Education level (n=629)  Secondary school or more  Primary school or less | *ref*  0.46 (0.27 - 0.78) | 0.004 | *ref*  0.80 (0.33 - 1.96) | 0.63 |
| Marital status (n=636)  Divorced/widow  Never married/single  Married/cohabiting | *ref*  0.13 (0.02 - 0.66)  0.34 (0.09 - 1.31) | 0.01  0.014  0.12 | *ref*  0.60 (0.36 - 3.05)  0.87 (0.68 – 7.17) | 0.19  0.34  0.56 |
| Source of income (n=636)  Casual work  Farming  Permanent work  Business | *ref*  1.51 (0.33 - 6.98)  2.53 (0.54 - 11.93)  1.29 (0.29 - 5.67) | 0.71  0.60  0.24  0.74 | *ref*  0.46 (0.09 - 2.54)  0.69 (0.12 - 3.98)  0.39 (0.08 - 1.87) | 0.36  0.38  0.68  0.24 |
| Religion (n=636)  Non-Christian  Christian | *ref*  1.07 (0.57 - 2.01) | 0.83 | *ref*  1.02 (0.37 - 2.82) | 0.97 |
| Gravidity (n=635)  Multi-gravida  Primi-gravida | *ref*  1.33 (0.78 - 2.26) | 0.30 | *ref*  1.29 (0.54 - 3.11) | 0.57 |
| History of miscarriage (n=635)  No  Yes | *ref*  0.67 (0.20 - 2.24) | 0.52 | *ref*  1.43 (0.32 - 6.36) | 0.64 |
| Maternal HIV status (n=636)  Sero-negative  Sero-positive | *ref*  1.19 (0.58 - 2.45) | 0.63 | *ref*  3.16 (1.24 - 8.06) | 0.016 |
| During child birth | | | | |
| Pregnancy outcome (n=632)  Poor outcome  Good outcome | *ref*  0.25 (0.09 - 0.66) | 0.006 | 0.09 (0.03 - 0.26) | <0.001 |
| Mode of birth (n=632)  Vaginal delivery  Caesarian section | *ref*  1.01 (0.44 - 2.31) | 0.98 | 2.67 (0.35 - 20.21) | 0.34 |
| Complication at childbirth (n=632)  No  Yes | *ref*  0.68 (0.34 - 1.39) | 0.29 | 0.17 (0.02 - 1.31) | 0.34 |
| Postpartum hemorrhage (n=632)  No  Yes | *ref*  1.58 (0.34 - 7.24) | 0.55 | 1.25 (0.57 – 10.92) | 0.76 |
| Sex of newborn (n=634)  Female  Male | *ref*  1.15 (0.68 - 1.95) | 0.61 | 1.97 (0.78 - 4.94) | 0.15 |
| Birth weight (n=634)  Normal birth weight  Low birth weight | *ref*  1.58 (0.80 - 3.09) | 0.19 | 1.97 (0.70 - 5.51) | 0.20 |
| Newborn admitted (n=635)  No  Yes | 1.05 (0.43 - 2.55) | 0.91 | 1.01 (0.23 - 4.44) | 0.99 |
| Maternal malaria status (n=633)  Malaria negative  Malaria positive | *ref*  0.71 (0.16 - 3.07) | 0.65 | Omitted^1^ |  |
| Maternal anaemia status (n=633)  Non-anaemic (Hb≥11g/dl)  Anaemic (Hb <11g/dl) | *ref*  2.24 (1.30 - 3.87) | 0.004 | *ref*  2.79 (1.16 - 6.71) | 0.022 |
| Maternal ID status (n=440)  Non ID  ID | *ref*  1.58 (0.80 - 3.10) | 0.23 | *ref*  0.85 (0.23 - 3.18) | 0.80 |
| Maternal IDA status (n=439)  Non IDA  IDA | *ref*  1.73 (0.68 - 4.41) | 0.25 | 1.44 (0.31 - 6.77) | 0.64 |
| Maternal inflammation (n=440)  No Inflammation  inflammation | *ref*  1.03 (0.34 – 6.54) | 0.32 | 1.14 (0.67 – 5.88) | 0.41 |
| CI: *confidence interval*  Good outcome: *live birth either full term or preterm birth*  Poor outcome: *stillbirth, spontaneous abortion, elective or induced abortion*  Early PPD: *postpartum depression measured between 24 to 72 hours after child birth*  EPDS*: Edinburg Postpartum Depression Scale*  ID: *iron deficiency indicated as serum ferritin<15 ug/L or ferritin <30 ug/L if C-reactive protein >5 μg/L*  IDA: *iron deficiency anaemia indicated as Hb<11g/dL and serum ferritin<15 ug/L or* *ferritin <30 ug/L if C-reactive protein >5 μg/L.* Inflammation: *indicates C-reactive protein >5μg/L,*  ref: *reference group*  ^1^: *omitted as a result of zero case* | | | | |
